# Time-resolved amino acid uptake of *Clostridium difficile* 630Δerm and concomitant fermentation product and toxin formation

**DOI:** 10.1186/s12866-015-0614-2

**Published:** 2015-12-18

**Authors:** Meina Neumann-Schaal, Julia Danielle Hofmann, Sabine Eva Will, Dietmar Schomburg

**Affiliations:** Technische Universität Braunschweig, Department of Bioinformatics and Biochemistry and Braunschweig Integrated Center of Systems Biology (BRICS), Langer Kamp 19b, 38106 Braunschweig, Germany

**Keywords:** *Clostridium difficile*, Metabolism, Stickland reaction, Amino acids, Fermentation products, Toxin

## Abstract

**Background:**

*Clostridium difficile* is one of the major nosocomial threats causing severe gastrointestinal infections. Compared to the well documented clinical symptoms, little is known about the processes in the bacterial cell like the regulation and activity of metabolic pathways. In this study, we present time-resolved and global data of extracellular substrates and products. In a second part, we focus on the correlation of fermentation products and substrate uptake with toxin production.

**Results:**

Formation of different fermentation products during growth in a comparison between the two different media in a global approach was studied using non-targeted gas chromatography–mass spectrometry (GC-MS) based analysis. During cultivation in a casamino acids medium and minimal medium, the clinical isolate *C. difficile* 630Δerm showed major differences in amino acid utilization: In casamino acids medium, *C. difficile* preferred proline, leucine and cysteine as carbon and energy sources while glutamate and lysine were not or hardly used. In contrast, proline and leucine were consumed at a significantly later stage in minimal medium. Due to the more complex substrate mixture more fermentation products were detectable in the casamino acids medium, accompanied by major changes in the ratios between oxidative and reductive Stickland products. Different glucose consumption dynamics were observed in presence of either casamino acids or the minimal set of amino acids, accompanied by major changes in butanoate formation. This was associated with a variation in both the toxin yield and a change in the ratio of toxin A to toxin B.

**Conclusions:**

Since in all media compositions, more than one substrate was available as a suitable carbon source, availability of different carbon sources and their metabolic fate appears to be the key factor for toxin formation.

**Electronic supplementary material:**

The online version of this article (doi:10.1186/s12866-015-0614-2) contains supplementary material, which is available to authorized users.

## Background

*Clostridium difficile* is one of the major nosocomial pathogens and causes antibiotic-associated diarrhoea and pseudomembranous colitis through its toxins. The major risk factor for a *C. difficile* infection is the exposure to antibiotics. The normal microflora of the gut is destroyed by the administration especially of broad-spectrum antibiotics allowing colonization and growth of *C. difficile* [1, 2].

The strain *C. difficile* 630Δerm used in this study was constructed by Hussain et al. [3] and is based on the strain 630 isolated during an outbreak in Switzerland and was first described in 1983 [4]. Toxin production is associated with dramatic changes in the bacterial metabolism and is linked to availability of substrates such as glucose, cysteine or proline [5]. Thus, detailed understanding of its metabolism and substrate preferences is an essential perquisite for the development of new therapeutic strategies.

**Fig. 1:**
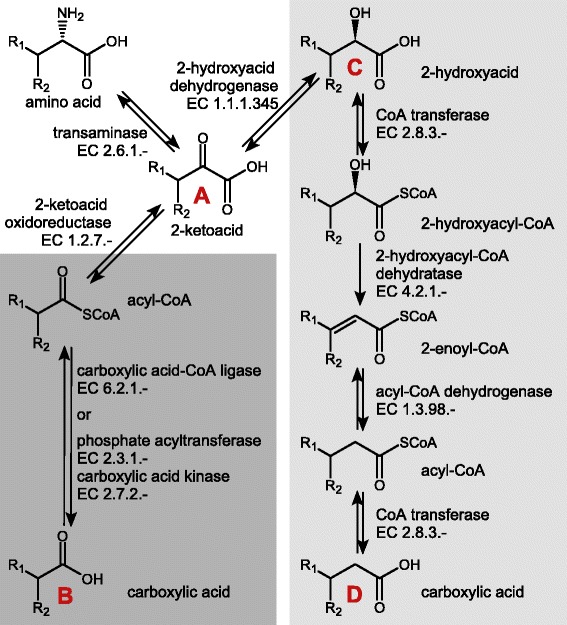
Generalized view of Stickland reactions. Light grey panel: reductive pathway, dark grey panel: oxidative pathway, R depending on the amino acid. Products and intermediates that may be detectable in the supernatant and are thus discussed below are labelled with red letters

Similar to a number of anaerobic bacteria, *C. difficile* has developed specific pathways to degrade amino acids and sugars by fermentation processes [6–8]. Due to the absence of any respiratory system, in this process energy is conserved mainly by substrate-level phosphorylation. Fermentation of amino acids is summarized under the name “Stickland reactions” involving the coupled oxidation and reduction of amino acids to their corresponding organic acids [9]. The electron donor amino acid is oxidized to a carboxylic acid one carbon atom shorter than the original amino acid while the electron acceptor amino acid is reduced to the corresponding deaminated carboxylic acid with the same length as the original amino acid. Specific amino acids can act as Stickland acceptors (reductive pathway, Fig. 1, light grey), Stickland donors (oxidative pathway, Fig. 1, dark grey), or as both. Some amino acids are known to follow modified pathways e.g. proline and glycine [10–13]. Early studies with different *C. difficile* isolates revealed its ability to produce Stickland products e.g. from leucine, isoleucine, valine, phenylalanine and tyrosine [6, 7]. On enzymatic level, Stickland reactions were studied in *C. difficile* with a special focus on leucine [14, 15]. More extensive studies were performed on *C. sticklandii* and *C. sporogenes* focussing on aromatic amino acids [16, 17].

Toxigenic *C. difficile* strains produce up to three major toxins [18]. Toxins A and B are members of the large clostridial toxin family which comprises various toxins from different *Clostridia* [19]. A third toxin is described as the CDT binary toxin, however it is not present in the strain 630 [20]. Toxin A and B are large single-chain mono-glucosyltransferases catalyzing the glycosylation and thereby the inactivation of Rho-GTPases leading to cell death [19, 21]. In addition to the genes of the toxins themselves, the genome of toxigenic *C. difficile* includes related genes encoding the positive regulator TcdR essential for toxin expression [22, 23], the antagonist TcdC [24] and the holin-like protein TcdE required for release from the cell [25].

While influence on growth and toxin formation of single substrates added to complex media is characterized [5–7, 26, 27], only a small number of studies focus on *C. difficile* grown in defined and minimal media. First studies on defined and minimal medium were performed by Haslam et al. [28] and Karasawa et al. [29]. All authors described major differences between different clinical and ecological isolates in deprivation experiments in media containing 18 amino acids: while all tested strain require isoleucine, leucine, valine and proline for growth, only some of them show detectable growth in the absence of either tryptophan [29], methionine, or cysteine [29]. Arginine, glycine, histidine and threonine were classified as growth-enhancing amino acids [29]. Minimal requirements for growth were defined by Karlsson et al. [30]. Jackson et al. [13] could show that L-4-hydroxyproline can replace L-proline in defined medium as a Stickland acceptor. However, hydroxyproline is not a direct substrate for D-proline reductase, so an intracellular conversion of hydroxyproline to proline via a yet unclear way is required. Addition of certain amino acids such as proline and glycine were only enhancing growth addition in presence of selenium due to the involvement of selenoenzymes, e.g. proline reductase and glycine reductase, in the catabolism of these amino acids.

Here, we present the first time-resolved and global study of consumption and export of metabolites involved in *C. difficile* 630Δerm fermentation in a defined casamino acid containing medium and in a minimal medium including a detailed view on metabolism-dependent toxin production. We give a detailed insight into the formation of different fermentation products during growth in a comparison between the two different media in a global approach using non-targeted gas chromatography–mass spectrometry (GC-MS) based analysis.

## Results and discussion

### Growth curve and glucose consumption in different media

**Fig. 2:**
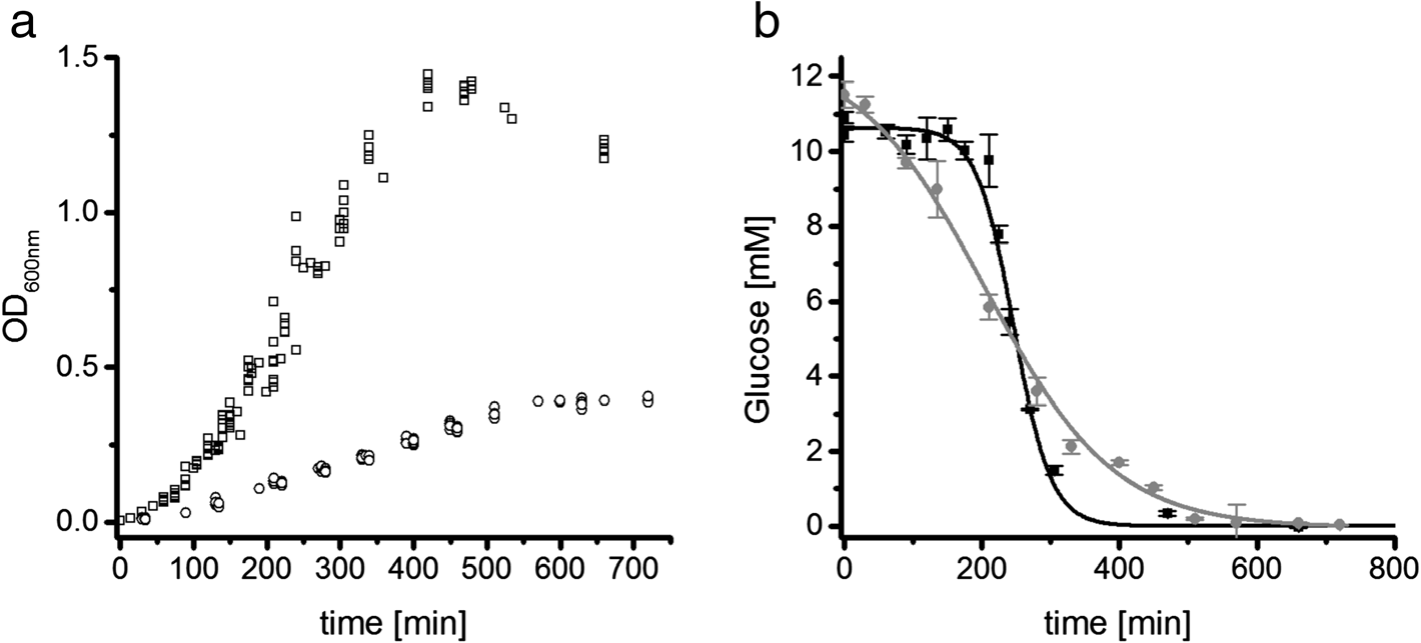
Growth curves and glucose consumption of *C. difficile* 630Δerm on CDMM and MDM. **a** Optical densities were measured at 600 nm of at least 10 different biological replicates; squares represent growth in CDMM, circles in MDM. **b** Glucose concentration in the culture supernatant after growth in different media. Concentration was determined enzymatically as described in Methods, squares/black line represent concentrations in CDMM, circles/grey line in MDM, curves were fitted according to the Boltzmann model using Origin9.0G software

*C. difficile* 630Δerm [3] showed a significantly altered growth in the casamino acids containing *C. difficile Minimal Medium* (CDMM) compared to *Minimal Defined Medium* (MDM) (Fig. 2a). While growth in CDMM showed a clear exponential growth phase, growth in MDM appeared to be more linear. Fitting of the growth curves revealed doubling times of 29.7 min and 48.3 min, respectively. The maximal optical density (OD_600nm_) differed from 1.4 in CDMM to 0.45 in MDM. Both media contained 2 g/L of glucose and 4.63 g/L and 3.6 g/L of amino acid mixtures, respectively (Tables 1 and 2). A significant pH shift was not observed in any culture (pH 6–7 at the end of cultivation). So, amino acid composition appeared to be crucial for the observed biomass yield. Based on the genome annotation, in addition to glucose only cysteine is available for biomass formation in MDM. Other amino acids in this medium, such as branched chain amino acids, tryptophan and proline, are most likely only used as energy source in Stickland reactions and for protein biosynthesis since *C. difficile* lacks appropriate degradation pathways connected to the central carbon metabolism [20, 31]. A similar energy metabolism was also observed for related Clostridia such as *C. sticklandii* [16]. More experimental data supporting this speculation are summarized in the chapter *Time-resolved amino acid uptake*.

**Table 1:** Substrate uptake of amino acids and glucose supplied in CDMM

Substrate	Initial concentration	Relative abundance in stationary phase	t_halfmaximal comsumption_	Maximal relative uptake rate
	g/L	%	min	h^−1^
Glucose	2.0	bdl^a^	246 ± 3	0.61 ± 0.07
Proline	0.36	bdl	106 ± 4	0.32 ± 0.02
Cysteine	0.5	bdl	124 ± 4	0.43 ± 0.03
Aspartate	0.27	16.3 ± 0.9	141 ± 8	0.34 ± 0.04
Arginine	0.14	35.7 ± 0.9	148 ± 7	0.20 ± 0.02
Alanine	0.16	19.4 ± 2.8	157 ± 11	0.23 ± 0.02
Leucine	0.38	bdl	161 ± 4	0.49 ± 0.08
Glycine	0.05	bdl	163 ± 7	0.31 ± 0.02
Tryptophan	0.1	45.7 ± 1.0	167 ± 15	0.12 ± 0.01
Serine	0.25	bdl	167 ± 2	0.62 ± 0.03
Isoleucine	0.24	bdl	170 ± 5	0.33 ± 0.02
Methionine	0.12	bdl	170 ± 7	0.45 ± 0.03
Tyrosine	0.24	25.7 ± 0.6	171 ± 17	0.19 ± 0.02
Threonine	0.21	bdl	177 ± 18	0.21 ± 0.01
Phenylalanine	0.20	bdl	177 ± 4	0.44 ± 0.01
Valine	0.27	5.4 ± 0.3	224 ± 13	0.24 ± 0.03
Lysine	0.34	101.0 ± 2.1	No significant consumption	
Glutamate	0.80	97.6 ± 3.9	No significant consumption	

Composition of casamino acids is based on distribution of amino acids in casein and was confirmed by enzymatic assays (for detailed procedure see Additional file 4); total sampling range up to 660 min, for detailed fits, see Additional file 1; ^a^bdl, below detection limit

**Table 2:** Substrate uptake of amino acids and glucose supplied in MDM

Substrate	Initial concentration	Relative abundance in stationary phase	t_halfmaximal comsumption_	Maximal relative uptake rate
	g/L	%	min	h^−1^
Glucose	2.0	bdl^a^	197 ± 10	0.17 ± 0.01
Methionine	0.2	bdl	136 ± 17	0.23 ± 0.04
Cysteine	2.0	bdl	164 ± 13	0.21 ± 0.01
Isoleucine	0.3	18.4 ± 0.8	306 ± 18	0.11 ± 0.01
Tryptophan	0.1	bdl	307 ± 23	0.13 ± 0.01
Proline	2.0	bdl	318 ± 4	0.56 ± 0.05
Leucine	0.4	27.2 ± 0.2	332 ± 8	0.16 ± 0.01
Valine	0.3	36.3 ± 0.9	347 ± 8	0.18 ± 0.02

Total sampling range up to 720 min, for detailed fits, see Additional file 2; ^a^bdl, below detection limit

Glucose was completely consumed in both, MDM and CDMM, but the dynamics differed significantly (Fig. 2b). Omitting glucose from the medium led to a significantly reduced OD_max_ of 0.16 ± 0.02 in MDM but not in CDMM. In CDMM no significant consumption of glucose was observed until the end of exponential growth followed by a rapid uptake. By the end of the transition phase, glucose concentrations were already below detection limit. In MDM, glucose was consumed over the whole growth curve with only 27 % of the maximal uptake rate compared to CDMM (Tables 1 and 2, Additional files 1 and 2).

### Time-resolved amino acid uptake

*C. difficile* showed a very complex preference for the different amino acids in the two media used. The most interesting result is the fact that out of the amino acids available in CDMM, the most abundant one, glutamate, was not used despite its internal important role as an amino-group transferring agent and its internal high concentration. On the other hand, the fact that aspartate was used indicates a high specificity of the aspartate/glutamate-transporter (CD630_25410) for aspartate, considering the high glutamate concentration in the medium (Table 1, Additional file 1) (compare also [27]). Lysine was also not utilized.

The import of the other available amino acids is highly diverse with rapidly imported ones (Ser, Met, Leu, Pro), amino acids with 50 % consumption in the middle of the exponential growth phase (Gly, Ile, Phe, Thr) and less efficiently used ones (Trp, Arg, Tyr, Ala, Asp, Val) (Table 1, Additional file 1). Independent from the overall rate of consumption most of the amino acids are imported from the start of the growth phase whereas valine was only slowly consumed in the beginning and much quicker later when rapidly used substrates were below detection limit. Apparently, valine is not a preferred substrate though it is subjected to oxidative Stickland fermentation and essential for *C. difficile* 630Δerm.

In the MDM only 7 amino acids are available for growth (Pro, Cys, Met, Val, Leu, Ile, Trp). Due to conflicting evidence obtained in *C. difficile* isolates in former deprivation studies [28, 29], we tested the essentiality of each of the seven amino acids. Except for a minimal growth observed in the absence of methionine, no growth was observed in the absence of each of the others (data not shown).

In time-dependent studies, cysteine and methionine were consumed from the start of growth. Proline was consumed only slowly in the beginning with a rapid consumption starting in the middle of the exponential phase (Table 2, Additional file 2). As in CDMM, tryptophan was consumed almost linearly during the whole growth.

Different preferences were observed for branched chain amino acids: while a clear order of preferences in CDMM was observable (leucine, isoleucine, valine), the three consumption curves were quite similar in MDM. None of them was consumed completely (Table 2, Additional file 2). This indicates that the entry into the stationary phase is not caused by a lack of energy sources but by the lack of a carbon source usable for biomass production. To verify this assumption, we added threonine solution to the culture at the beginning of the stationary phase which resulted in a restart of growth. The presence of branched chain amino acids has been previously shown to influence DNA-binding affinities of the global regulator CodY so that consumption of these amino acids is supposed to have major influences on the enzyme repertoir and thereby on the metabolism [32, 33].

*C. difficile* shares a high genomic similarity with *C. sticklandii*, and thus a similar energy metabolism was assumed in the literature [16] but in this study we could show significant differences in amino acid utilization and fermentation product formation. Secretion of alanine was only observed in MDM but not in medium with more amino acids (CDMM) and no secretion of glutamate or aspartate was detected. Aspartate was actually consumed by *C. difficile*. Branched chain amino acids, especially leucine are preferred substrates of *C. difficile* but not of *C. sticklandii.* Lysine is consumed by *C. sticklandii* at a late stage of growth, but not by *C. difficile* under our growth conditions.

### Metabolite export during growth

Analogous to substrate consumption, a defined order was observed for fermentation product formation. Overall, we could detect 25 different products exported during growth in CDMM (Table 3, Additional file 3). For some products the origin is obvious: as the reductive Stickland products (Fig. 1d), 5-aminovalerate was produced from proline, isocaproate from leucine and 3-phenylpropanoate from phenylalanine. The product of tyrosine 3-(4-hydroxyphenyl)propanoate was detectable only in traces (data not shown). Additional oxidative Stickland products (Fig. 1b) occurred in CDMM: isovalerate produced from leucine, (4-hydroxyphenyl)acetate from tyrosine, 2-methylbutanoate from isoleucine, isobutanoate from valine and phenylacetate from phenylalanine. Also some intermediates of the Stickland reactions were detectable in the supernatant: 2-oxo-isocaproate (Fig. 1a), 3-(4-hydroxyphenyl)lactate and 3-phenyllactate (both Fig. 1c). This is surprising since it is not energetically favourable. This is possibly due to either an imbalance of the oxidative and reductive pathways or an unspecific export of the intermediates. A number of fermentation products is produced via acetyl-CoA and/or propanoyl-CoA: acetate, valerate, propanoate, butanoate, and 3-hydroxybutanoate. Acetyl-CoA is formed from several sources, e.g. threonine, glycine, serine, alanine and glucose. Propanoyl-CoA is produced from e.g. threonine and methionine. Later in growth, also different alcohols occurred in the supernatant (Table 3).

**Table 3:** Exported metabolites detected in growth with CDMM

Product	t_first detection_	t_halfmaximal production_	Maximal relative production rate
	min	min	h^−1^
Isovalerate	60	83 ± 5	0.59 ± 0.02
Propanoate	80	120 ± 8	0.39 ± 0.01
2-oxo-isocaproate	90	155 ± 12	0.26 ± 0.08
(4-hydroxyphenyl)acetate	150	161 ± 12	0.39 ± 0.04
5-aminovalerate	60	163 ± 7	0.33 ± 0.03
2-methylbutanoate	60	168 ± 13	0.45 ± 0.05
Acetate	90	175 ± 6	0.30 ± 0.02
3-(4-hydroxyphenyl)lactate	175	181 ± 5	0.78 ± 0.14
Isocaproate	60	194 ± 7	0.47 ± 0.05
Isobutanoate	60	202 ± 5	0.59 ± 0.04
Ammonium	traces in medium	210 ± 28	0.19 ± 0.04
2-aminobutanoate	150	213 ± 6	0.28 ± 0.02
Formate	60	228 ± 10	0.24 ± 0.01
Phenylacetate	60	231 ± 4	0.38 ± 0.03
Ethanol	210	259 ± 6	0.32 ± 0.04
3-hydroxybutanoate	240	261 ± 7	0.62 ± 0.13
p-cresol	60	263 ± 9	0.26 ± 0.02
2-hydroxybutanoate	150	273 ± 4	0.30 ± 0.03
1-butanol	225	274 ± 9	0.39 ± 0.03
Isobutanol	240	276 ± 8	0.46 ± 0.03
Butanoate	175	285 ± 6	0.30 ± 0.04
Valerate	175	315 ± 4	0.25 ± 0.06
3-phenylpropanoate	175	355 ± 9	0.21 ± 0.06
Lactate	225	364 ± 3	0.27 ± 0.02
3-phenyllactate	120	387 ± 21	0.12 ± 0.01

All compounds with the exception of formate and ammonium were detected by GC-MS; formate and ammonium were quantified by enzymatic assays (see Methods), total sampling range up to 660 min, for detailed fits, see Additional file 3

2-Aminobutanoate and 2-hydroxybutanoate originate from the intermediate 2-oxobutanoate, which occurs in threonine and methionine degradation, both were first detectable at the same time of the growth curve. 2-Aminobutanoate could be one form of exporting excess nitrogen from the cell. Additionally, ammonium was found in the supernatant released via deamination from all amino acids except for proline, leading to an ammonium formation in both MDM and CDMM (Tables 3 and 4). The formation of 2-aminobutanoate was described earlier for *C. difficile* in complex media [8]. Formate was produced either via 2-oxobutanoate or from pyruvate by formate-C-acetyltransferase (EC 2.3.1.54) yielding propanoyl-CoA or acetyl-CoA, respectively [31].

**Table 4:** Product formation in MDM

Product	t_first detection_	t_halfmaximal production_	Maximal relative production rate
	min	min	h^−1^
Formate	30	202 ± 18	0.16 ± 0.02
2-hydroxyisocaproate	135	225 ± 12	0.21 ± 0.02
Isocaproate	90	239 ± 14	0.14 ± 0.01
Isobutanoate	135	258 ± 39	0.12 ± 0.02
Isovalerate	30	280 ± 9	0.13 ± 0.02
2-methylbutanoate	30	290 ± 43	0.11 ± 0.01
5-aminovalerate	210	360 ± 8	0.21 ± 0.02
2-oxoisocaproate	280	435 ± 16	0.15 ± 0.02
Alanine	280	442 ± 18	0.18 ± 0.01
Acetate	280	457 ± 20	0.12 ± 0.01
Ammonium	traces in medium	470 ± 49	0.11 ± 0.01

All compounds with the exception of formate and ammonium were detected by GC-MS; formate and ammonium were quantified by enzymatic assays (see Methods), total sampling range up to 720 min, for detailed fits, see Additional file 2

In the MDM-culture, the fermentation product profile was less complex (Table 4, Additional file 2). As in CDMM, 2-methylbutanoate, isobutanoate, isocaproate and isovalerate were produced from branched chain amino acids and 5-aminovalerate from proline. As intermediates 2-oxo-isocaproate (Fig. 1a) and additionally 2-hydroxyisocaproate (Fig. 1c) were detectable. In the stationary phase, also low amounts of 2-oxo-isovalerate and 3-methyl-2-oxovalerate were detectable corresponding to the 2-ketoacids derived from valine and isoleucine (Fig. 1a).

In contrast to CDMM, no butanoate formation was observed in MDM. This is in accordance with the fact that in CDMM more sources for biomass formation are available (e.g. threonine, serine, glycine). Accordingly, also short-chain alcohol formation was not detectable in the MDM-culture.

Moreover, qualitative analysis showed that both sulfite and sulfide were produced in MDM and CDMM (data not shown).

### Time-dependent leucine fermentation in CDMM and MDM

**Fig. 3:**
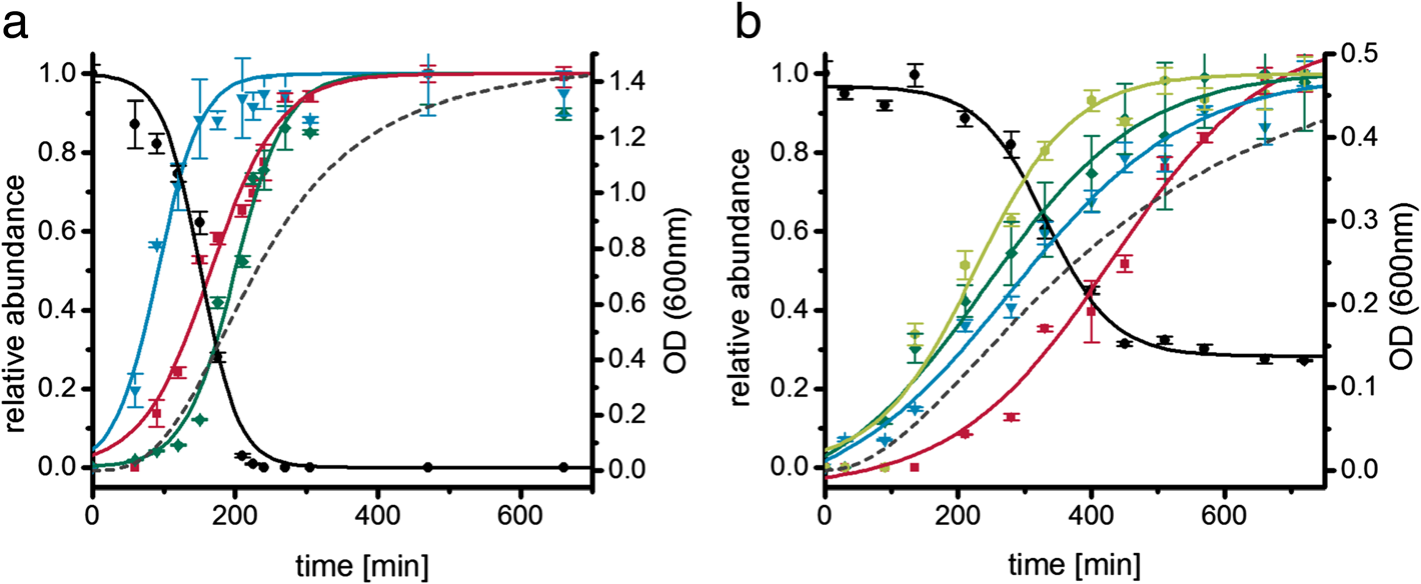
Leucine fermentation of *C. difficile* 630Δerm on CDMM and MDM. Left axis: black circles, leucine; blue triangles, isovalerate; red squares, 2-oxo-isocaproate; green rhombs, isocaproate; light green octagons, 2-hydroxyisocaproate; right axis: dotted line, growth curve; curves were fitted according to the Boltzmann model using Origin9.0G software. **a** Leucine consumption and fermentation product formation in CDMM. **b** Leucine consumption and fermentation product formation in MDM

Leucine as a Stickland amino acid is of special interest since it can be metabolized by the oxidative and reductive pathway [14, 15]. In the first growth phase the major exported product from leucine was isovalerate (Fig. 3a). Then the intermediate of both pathways, 2-oxo-isocaproate was found in the supernatant before the reductive product isocaproate was detectable in the supernatant. Maximal isovalerate concentration was already reached at a stage where 25 % of the initial leucine was still available. Though isocaproate was also detectable in the early growth phase, major isocaproate production started when the concentrations of other substrates for reductive fermentation such as proline and glycine concentrations were already significantly reduced (25 % and 50 %, respectively). In MDM, no clear temporal separation was observable (Fig. 3b): isocaproate and isovalerate were exported simultaneously. In contrast to CDMM, the reductive intermediate 2-hydroxyisocaproate was detectable during growth in MDM. 2-oxo-isocaproate was also detectable but exported later in growth compared to isocaproate.

**Fig. 4:**
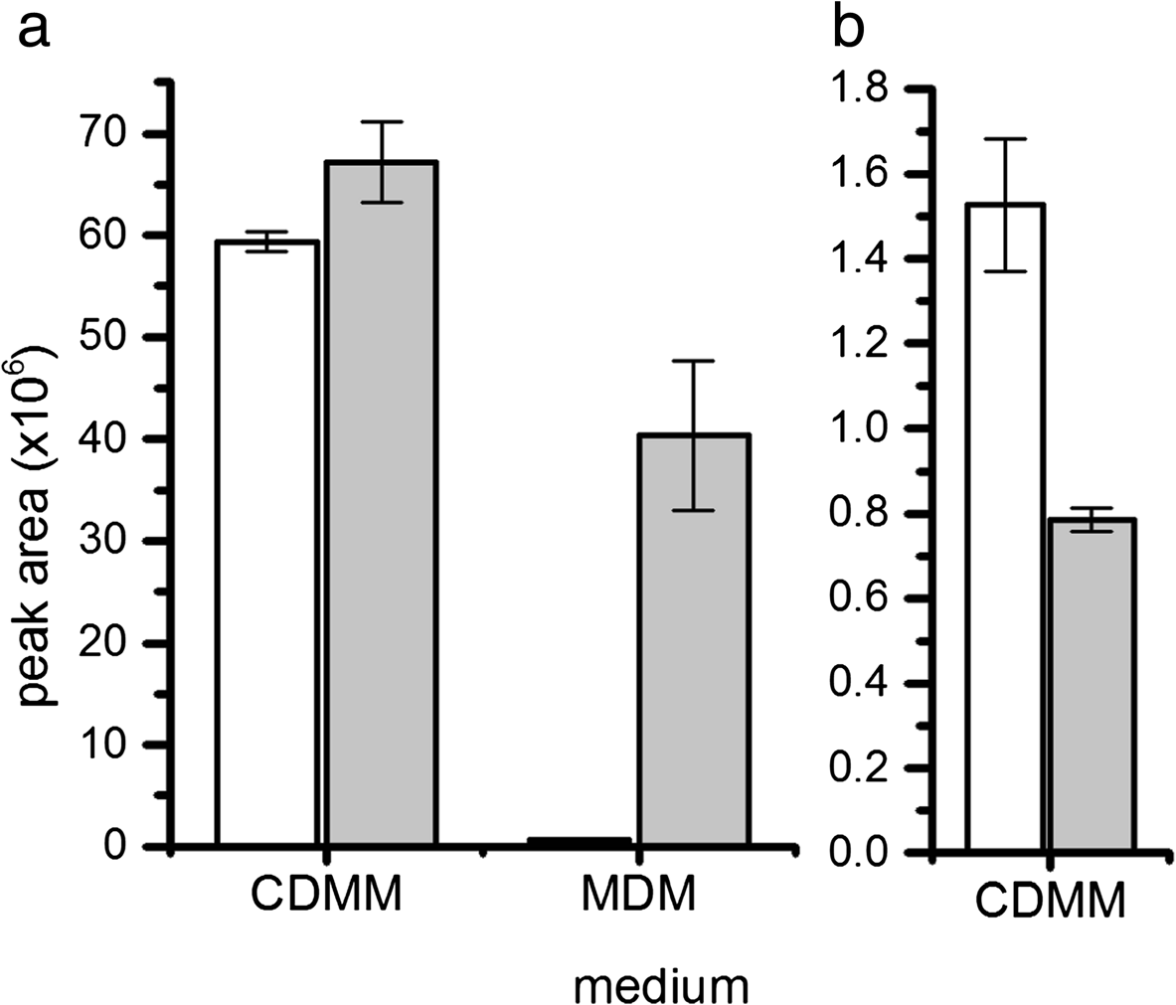
Detected relative amounts of leucine and phenylalanine fermentation products in stationary phase. **a** White bars: isocaproate, grey bars: isovalerate. Determined in non-derivatized samples after ether extraction by GC/MS, peak areas were determined using MetaboliteDetector software and were normalized on the relative proportion of the specific quantification ion and the internal standard o-cresol added prior to extraction. **b** White bar, 3-phenylpropanoate, grey bar, phenylacetate. Determined in derivatized samples after drying by GC/MS. Peak areas were determined using MetaboliteDetector software and were normalized on ribitol and the relative proportion of the specific quantification ion added as internal standard prior to drying procedure. We cannot exclude differences of the two compounds during extraction, drying or derivatization procedures

While isocaproate and isovalerate showed a comparable abundance in chromatograms obtained from samples in CDMM cultures (Fig. 4a), the most abundant fermentation product in MDM was isovalerate with the peak area of isocaproate being only 2 % of that from isovalerate. The change between the different media was dramatic indicating that reductive fermentation in MDM was mainly covered by proline yielding 5-aminovalerate and that all exported isovalerate originated from leucine and not from valine.

This agrees with the enzyme characterization of the purified enzymes, involved in *C. difficile* leucine degradation, ((R)-2-hydroxyisocaproate dehydrogenase, (R)-2-hydroxyisocaproate-CoA transferase and 2-hydroxyisocaproyl-CoA dehydratase). The putative valine fermentation intermediate 2-oxoisovalerate was not accepted as a substrate by (R)-2-hydroxyisocaproate dehydrogenase [14, 15]. Moreover, we did not observe the putative product of isoleucine, 3-methylvalerate, in the supernatant.

### Time-dependent phenylalanine degradation in CDMM

**Fig. 5:**
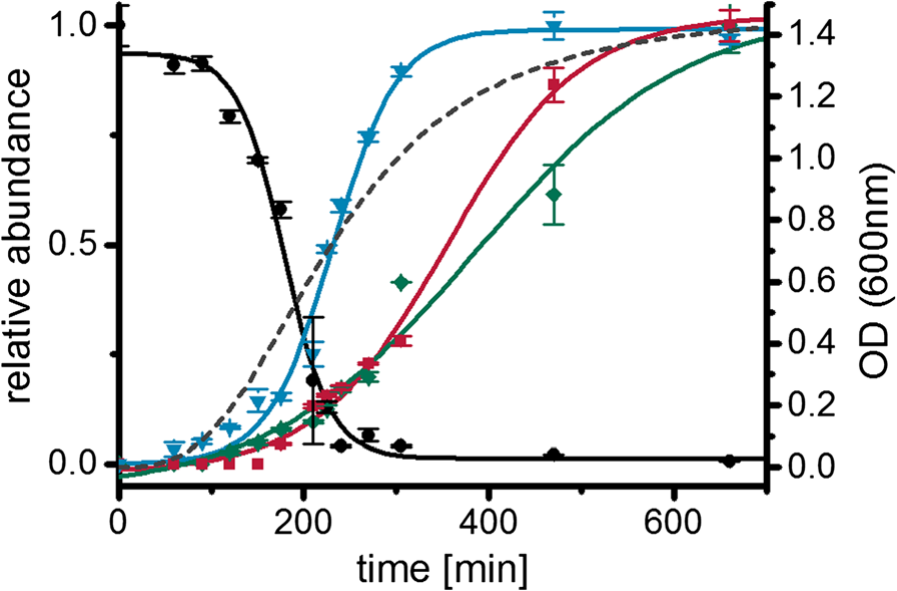
Phenylalanine fermentation of *C. difficile* 630Δerm on CDMM. black circles, phenylalanine; blue triangles, phenylacetate; red squares, 3-phenylpropanoate; green rhombs, 3-phenyllactate; dotted line, growth curve; curves were fitted according to the Boltzmann model using Origin9.0G software

Phenylalanine belongs also to those amino acids, that can be degraded via Stickland reaction, both oxidatively and reductively (Fig. 1). The growth curve revealed a preference for phenylalanine as a substrate for oxidative Stickland reaction. Phenylacetate was already detectable 60 min after the end of the lag-phase, i.e. at the beginning of the exponential growth phase (Fig. 5). The reductive intermediate 3-phenyllactate was detectable 60 min later and the final reductive product 3-phenylpropanoate 115 min later. Phenylalanine as both oxidative and reductive substrate is rather unusual among clostridia. For *C. sporogenes*, *C. botulinum*, and *C. sticklandii* only one of them, either phenylacetate or 3-phenylpropanoate as product was found [7, 34]. In an earlier study with a different *C. difficile* isolate in complex medium, 3-phenyllactate was not detected in the supernatant [7]. The detailed analysis of the total amounts accumulated in the supernatant in the stationary phase revealed that *C. difficile* produced both phenylalanine fermentation products in similar amounts with a tendency to higher amounts of the reductive product 3-phenylpropanoate (Fig. 4b).

*C. difficile* contains only one operon coding for genes of the classical reductive Stickland degradation (*ldhA* and *hadAIBC*, locus-tag CD630_03940 to _03980) [31]. Though the specific function was assigned to leucine degradation, earlier in vitro studies showed an activity of the (R)-2-hydroxyisocaproate dehydrogenase (LdhA) for phenylpyruvate (corresponding to Fig. 1a) [14, 15]. Thus, most likely aromatic acids are fermented with the same set of enzymes as leucine. Our data show that significant reductive phenylalanine fermentation was only observed when extracellular leucine levels dropped below 10 % of the initial concentration. As observed for leucine, the intermediate product 3-phenyllactate (Fig. 1c) was exported earlier than the final product. These data are in accordance with enzymatic in vitro data which revealed a higher affinity of the (R)-2-hydroxyisocaproate dehydrogenase to 2-oxo-isocaproate (the intermediate of leucine fermentation) compared to phenylpyruvate [15] and clearly support a physiological role for the metabolism not only of leucine but also of phenylalanine.

### Toxin formation in CDMM and MDM

**Fig. 6:**
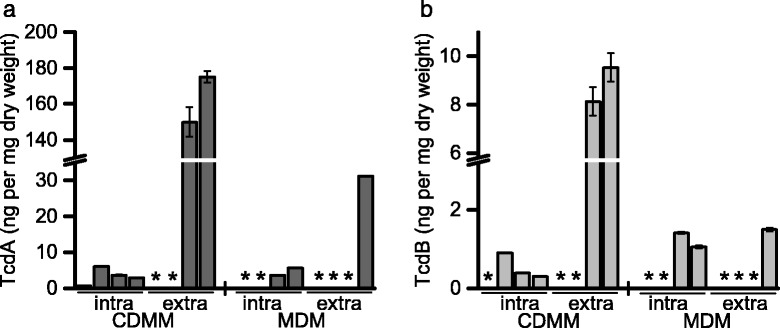
Toxin formation in CDMM and MDM. Toxin A (**a**, dark grey bars) and Toxin B (**b**, light grey bars) was determined in culture supernatants (extra) and intracellularly (intra) in the exponential, transient and early stationary phase and after 48 h of cultivation (from left to right) using an immunoassay and was calculated per mg of *C. difficile* dry weight, * below detection limit

Toxin formation is typically characterized in cultures 48–72 h after inoculation. Earlier during growth, toxin detection is possible rather intracellularly than in the supernatant [30]. Toxin formation was determined with an ELISA assay in the exponential, transient and early stationary phase and after 48 h of growth (Fig. 6). We did not observe detectable toxin concentrations extracellularly during the exponential and transient growth phase in CDMM and not until late stationary phase in MDM. Intracellularly, toxins were detectable earlier during growth in both media. In CDMM, Toxin A was quantified with 175.2 ± 3.2 ng and Toxin B with 10.0 ± 0.6 ng exported per mg of dry biomass until late stationary phase. Toxin A was quantified with 31.17 ± 0.06 ng and Toxin B with 1.53 ± 0.05 ng exported per mg of dry biomass in MDM. The ratio Toxin A to Toxin B was 18 in CDMM compared to 20 in MDM.

Toxin production varies dramatically between different isolates of *C. difficile* and different publications [30, 35, 36] and quantification of toxins depends on the quantification method and its feasibility of calibration. Comparison of the extracellular toxin level of five different strains including strain 630 revealed the lowest toxin scores for *C. difficile* 630 in a complex medium after 24 h cultivation with a toxin A to toxin B ratio of 1.4 [36]. Especially the ratio of Toxin A and Toxin B differs dramatically from our data obtained for *C. difficile* 630Δerm in defined media. Apparently, medium composition and tested growth phase influence not only the total amounts of toxin but also the ratio between toxin A and B. The change of ratio is especially interesting since toxin B is considered to be 100-1000-fold more cytotoxic than toxin A [19, 37].

While neither growth phase nor growth rate had a dramatic effect on toxin production of the strain VPI 10463, glucose limitation reduced toxin yields 20- to 100-fold in defined media [30]. Effects of glucose vary depending on the medium between a toxin-repressing effect in rich media and an enhancing one in defined media [26, 30]. Effects of specific amino acids are also largely depending on the basic medium as shown for cysteine and a mixture of nine amino acids [5, 30]. Among these nine amino acids were essential amino acids such as the branched chain amino acids, proline and tryptophan. Branched chain amino acids are known to increase the binding affinity of the global regulator CodY to the *tcdR* promotor region of the pathogenicity locus thereby repressing toxin synthesis in rich media [32, 33]. Earlier studies especially with *C. difficile* VPI 10463 revealed that different defined media with amino acid compositions comparable to MDM and CDMM had little influence on intracellular toxin formation [30]. Comparing the total toxin yield (intra- and extracellular), results were different: while a medium comparable to CDMM (MDM supplemented with threonine, glycine and casamino acids) yielded a much higher total toxin production ranging between the yields obtained in the complex PY and PYG media [30], MDM supplemented with threonine and glycine yielded significantly lower toxin yields [5]. Here, we observed a similar effect for strain 630Δerm. Moreover, we could show that not only the absolute concentration but also the ratio of both toxins is altered.

A significant interconnection between butanoate formation and toxin formation due to coupled regulation was discussed mainly for *C. difficile* strain VPI 10463 [5]. Bouillaut et al. speculated that this link may be a response to high intracellular NADH levels or to other gastrointestinal bacteria producing butanoate [38]. Our results show dramatic differences in the butanoate production between two defined media. In the absence of butanoate formation, we observed 5.6-fold lower toxin A and 6.5-fold lower toxin B formation despite the presence of equal amounts of glucose in the medium. A link to intracellular NADH levels is supported by the low toxin levels in MDM where leucine as one of preferred reductive substrates is still available in the stationary phase thereby preventing excess reducing equivalents. Basically, these data support a co-regulation of genes of toxin and butanoate formation but it clearly indicates that focussing on a single substrate itself is not an appropriate indicator for effects on toxin production.

## Conclusions

Our analysis of amino acid utilization demonstrated that *C. difficile* degrades amino acids in a highly complex, but defined way, depending on the composition of the medium. The data generated with casamino acids containing medium showed that proline, leucine and cysteine are the preferred sources of energy and carbon whereas glutamate and lysine are not or hardly used. Comparison of amino acid preferences in different media revealed that two of the preferred substrates in CDMM, proline and leucine were consumed in MDM at a later stage of growth while cysteine and methionine were consumed first. Our data show that *C. difficile* is optimal adapted to conditions observed in the intestine: Growth in more complex media such as CDMM is not dependent on monosaccharides as carbon source since their availability is limited in the large intestine [39]. *C. difficile* reaches high doubling rates almost comparable to respiring bacteria under appropriate conditions and is specialized on amino acids that are not so commonly used by other bacteria: like branched chain and aromatic amino acid as well as hydroxyproline. A typical and widely used amino acid such as glutamate is not a substrate of *C. difficile* (for a comparison with other human-pathogenic and environmental bacteria see [40–42]).

Due to the severe influence of amino acids on toxin formation, some authors suggested effects with regard to infection and therapy [28, 30, 43]. Our data suggest that toxin formation is less dependent on the presence of glucose in the medium than on its metabolic fate in *C. difficile* when different defined media were supplemented with glucose and cysteine. Since both cysteine and glucose are suitable carbon sources, availability of different carbon sources also in complex media could be one of the key factors for toxin formation. Thus, an isolated view on single substrates will not lead to a detailed understanding of the underlying regulation. Detailed and global analysis of the metabolic changes involved in toxin formation will give detailed insights into the metabolic fate of available substrates to establish a metabolic indicator useful for therapy development.

## Methods

### Strains, media and growth conditions

All studies were carried out with *C. difficile* 630Δerm (DSM28645) [3] obtained from the Deutsche Sammlung von Mikroorganismen und Zellkulturen (DSMZ, Braunschweig, Germany). Strain maintenance was performed routinely by growing the cells in anaerobized brain-heart-infusion medium in an anaerobic chamber (95 % N_2_/5 % H_2_) and cells were checked for antibiotic sensitivity on a regular basis.

CDMM is a defined medium containing glucose and casamino acids as carbon and energy sources and was prepared as described by Cartman and Minton [44] with following modifications: NaHCO_3_ was replaced by NaH_2_PO_4_ and a final concentration of 1 μM sodium hydrogenselenite was added (according to [13]), the glucose and biotin concentrations were reduced to 2 g/L and 0.012 mg/L, respectively (according to [30]) and the tryptophan concentration was reduced to 0.1 g/L. Casamino acids were obtained from Roth (Carl Roth GmbH, Karlsruhe, Germany). MDM contains seven essential amino acids (isoleucine, leucine, valine, tryptophan, cysteine, methionine and proline) and glucose as carbon and energy sources and was prepared as described by Karlsson et al. [30] with following modifications: NaHCO_3_ was replaced by NaH_2_PO_4_ and a final concentration of 1 μM sodium hydrogenselenite was added (according to [13]), cysteine and proline concentrations were increased to 2 g/L.

Cells were passaged twice with a dilution in MDM or CDMM as required of at least 1:100 prior to inoculation of the main culture.

Main cultures were inoculated at an OD_600nm_ of ~ 0.005 and growth at 37 °C was determined by following the optical density at 600 nm. Due to differences in the lag-phases of biological replicates, cultures were lag-phase corrected. Samples at different time points were taken anaerobically from at least 10 biological replicates. Samples for further analysis were taken throughout the whole growth curve.

### Enzymatic assays

L-amino acids (except glycine) were quantified using the L-Amino Acid Quantitation Kit (Sigma-Aldrich, St. Louis, MO, USA). Glucose, glutamate, formate and ammonium were assayed using R-biopharm yellow line kits (D-glucose, glutamic acid, formic acid and ammonia). Due to interference of supernatant components with the glucose assay, sample preparation prior to glucose quantification had to be modified. For the modified sample preparation, we included a drying step under vacuum at 50 °C and resolving the dried sample in water. Spiking experiments revealed a recovery of 91–99 % of added glucose.

Alanine was quantified by an enzymatic assay with L-alanine dehydrogenase from *Bacillus cereus* (Sigma-Aldrich, St. Louis, MO, USA) as described in [45] with the minor modifications. NADH formation was followed at 340 nm with 700 μL carbonate buffer pH 10, 100 μL of sample, 100 μL 20 mM NAD and 100 μL L-alanine dehydrogenase solution 0.5 U/mL for 5 min. For validation a 1 mg/mL L-Alanine was used whereby concentration ranges between 0.01 and 0.2 mg/mL.

The distribution of amino acids in casamino acids is based on the protein sequences of bovine casein [46]. The theoretical ratio between alanine and glutamate (0.2) was confirmed by enzymatic assays (0.2). The concentrations of the remaining amino acids were calculated based on the experimentally determined concentration of alanine and confirmed by determination of free L-amino acids (except glycine). Details are listed in Additional file 4.

### Toxin quantification

Toxins A and B were quantified using the TGC-E002-1 ELISA (tgcBIOMICS GmbH, Bingen, Germany) for the separate detection of Toxin A and Toxin B. For intracellular toxin levels cells were lysed according to [30]. The supernatant of harvested samples was used immediately.

### Gas chromatography/mass spectrometry based analysis of volatile compounds

The extraction and GC method was adapted from Su et al. [47] including several modifications: 1/5 volume of HPLC-grade sulphuric acid and o-cresol as internal standard were added to the culture supernatant and volatile compounds were extracted by vigorously mixing with 200 μL of *tert*-methylbutylether. After centrifugation at 4 °C, 10 min and 8000 g, the ether phase was transferred into a GC-MS vial and 1 μL of the extract was injected into a Thermo DSQ II gas chromatograph equipped with a liner and quadrupol mass spectrometer. Chromatography was carried with an Agilent VF-WAXms column (30 m length, 0.25 mm inner diameter, Agilent, Santa Clara, CA, USA) at a constant flow of 1 mL/min helium. The temperature program started at 55 °C held for 1 min, followed by temperature ramping of 10 °C min to a final temperature of 250 °C, which was held constant for 2 min. Solvent delay time was 2.4 min. A retention index marker (n-alcanes ranging from C10…C22 in cyclohexane) was used to convert retention times to retention indices.

### Gas chromatography/mass spectrometry based analysis of non-volatile compounds

10 μL of the supernatant was mixed with 500 μL ethanol spiked with 4 μg/mL of ribitol as internal standard. For further sample preparation, derivatization and measurement, samples were treated as described in Reimer et al. [48] with minor modifications: During the derivatization procedure the Gerstel MPS 2 XL Twister is equipped with a mVorx unit applied to resolve and mix the samples at 1300 rotations per min for 1 min both after addition of methoxyamine-pyridine and N-methyl-N-trimethylsilyltrifluoroacetamide.

### Data analysis

Raw data obtained from GC/MS measurements were processed by applying version 2.2 N-2013-01-15 of the in-house developed software MetaboliteDetector [49]. The peak identification was performed non-targeted with a combined compound library for each GC column applied. After processing, non-biological peaks and artefacts were eliminated by the aid of blanks. Peak areas were normalized to the corresponding internal standards (o-cresol or ribitol) and derivatives were summarized. Data were fitted sigmoidally after Boltzmann and uptake rates were determined using Origin9.0G software when applicable.

For leucine and phenylalanine fermentation products, peak areas were estimated after normalization on the relative proportion of the specific quantification ion compared to the total ion chromatogram but we cannot exclude differences of the two compounds during drying or derivatization procedures.

## Additional files

Additional file 1:
**Substrate consumption in CDMM.** Plotted were relative abundances based on peak areas (except glucose) were obtained by GC-MS or concentrations obtained by enzymatic assays (glucose) and fitted using OriginPro 9.0G software. (PDF 321 kb) Additional file 2:
**Substrate consumption and product formation in MDM.** Plotted were relative abundances based on peak areas (except glucose, formate and ammonium) were obtained by GC-MS or concentrations obtained by enzymatic assays (glucose, formate and ammonium) and fitted using OriginPro 9.0G software. (PDF 371 kb) Additional file 3:
**Product formation in CDMM.** Plotted were relative abundances based on peak areas (except formate and ammonium) were obtained by GC-MS or concentrations obtained by enzymatic assays (formate and ammonium) and fitted using OriginPro 9.0G software. (PDF 397 kb) Additional file 4:
**Casein composition and deduced amino acid composition of casamino acids.** Data were confirmed by enzymatic measurements of alanine, glutamate and free amino acid contents. (PDF 34 kb)

## Abbreviations

GC/MS: Gas chromatography–mass spectrometry
CDMM: *C. difficile* defined minimal medium
MDM: Minimal defined medium
OD: Optical density
