# Supplementary material for: Time-resolved amino acid uptake of Clostridium difficile 630Δerm and concomitant fermentation product and toxin formation
Source: BMC Microbiol. 2015 Dec 18;15:281. doi: 10.1186/s12866-015-0614-2 (PMC4683695; doi:10.1186/s12866-015-0614-2)
Supplement: Additional file 1: — Substrate consumption in CDMM. Plotted were relative abundances based on peak areas (except glucose) were obtained by GC-MS or concentrations obtained by enzymatic assays (glucose) and fitted using OriginPro 9.0G software. (PDF 321 kb) [file 12866_2015_614_MOESM1_ESM.pdf]

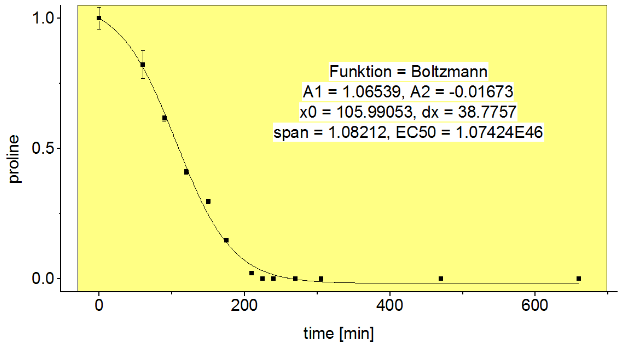
proline
1.0
0.5
0.0
0
200
400
600
time [min]
Funktion = Boltzmann
A1 = 1.06539, A2 = -0.01673
x0 = 105.99053, dx = 38.7757
span = 1.08212, EC50 = 1.07424E46

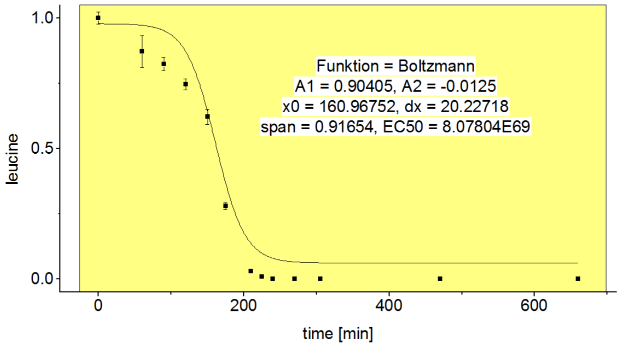
leucine
1.0
0.5
0.0
0
200
400
600
time [min]
Funktion = Boltzmann
A1 = 0.90405, A2 = -0.0125
x0 = 160.96752, dx = 20.22718
span = 0.91654, EC50 = 8.07804E69

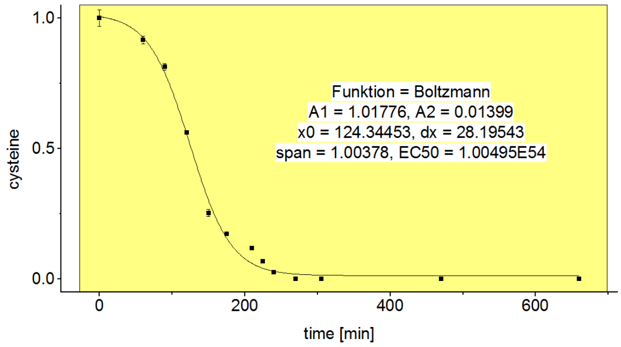
cysteine
1.0
0.5
0.0
0
200
400
600
time [min]
Funktion = Boltzmann
A1 = 1.01776, A2 = 0.01399
x0 = 124.34453, dx = 28.19543
span = 1.00378, EC50 = 1.00495E54

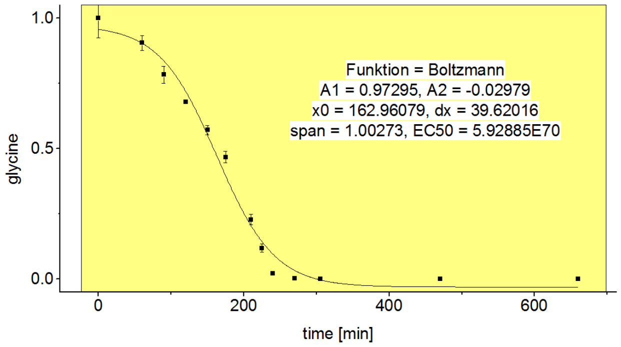
glycine
1.0
0.5
0.0
0
200
400
600
time [min]
Funktion = Boltzmann
A1 = 0.97295, A2 = -0.02979
x0 = 162.96079, dx = 39.62016
span = 1.00273, EC50 = 5.92885E70

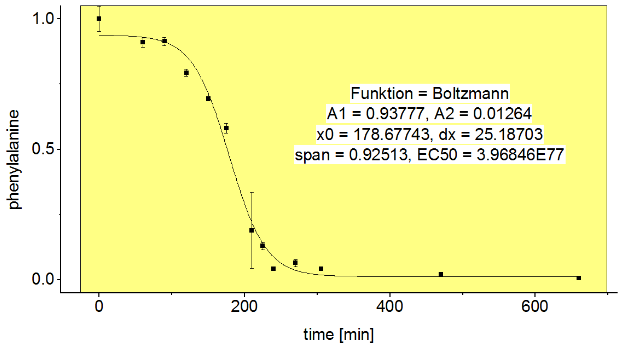
phenylalanine
1.0
0.5
0.0
0
200
400
600
time [min]
Funktion = Boltzmann
A1 = 0.93777, A2 = 0.01264
x0 = 178.67743, dx = 25.18703
span = 0.92513, EC50 = 3.96846E77

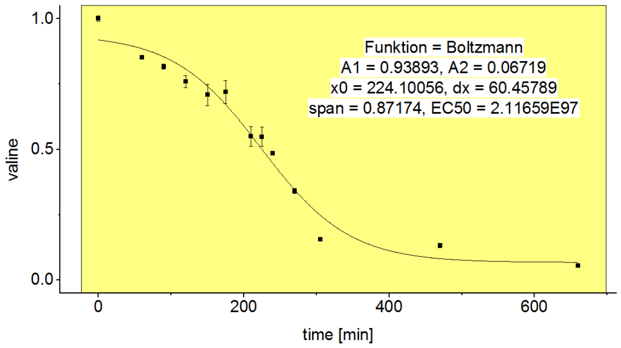
valine
1.0
0.5
0.0
0
200
400
600
time [min]
Funktion = Boltzmann
A1 = 0.93893, A2 = 0.06719
x0 = 224.10056, dx = 60.45789
span = 0.87174, EC50 = 2.11659E97

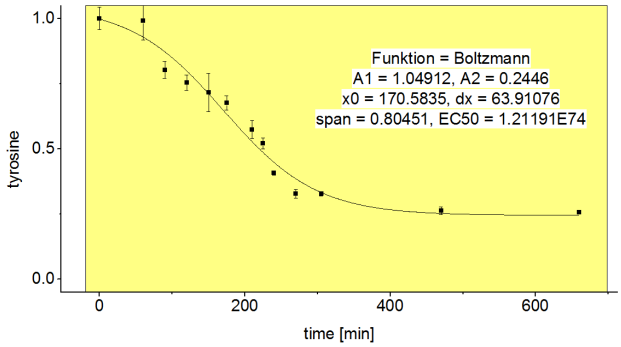
tyrosine
1.0
0.5
0.0
0
200
400
600
time [min]
Funktion = Boltzmann
A1 = 1.04912, A2 = 0.2446
x0 = 170.5835, dx = 63.91076
span = 0.80451, EC50 = 1.21191E74

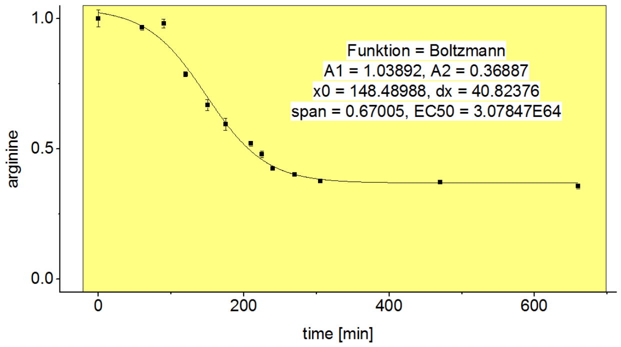
arginine
1.0
0.5
0.0
0
200
400
600
time [min]
Funktion = Boltzmann
A1 = 1.03892, A2 = 0.36887
x0 = 148.48988, dx = 40.82376
span = 0.67005, EC50 = 3.07847E64

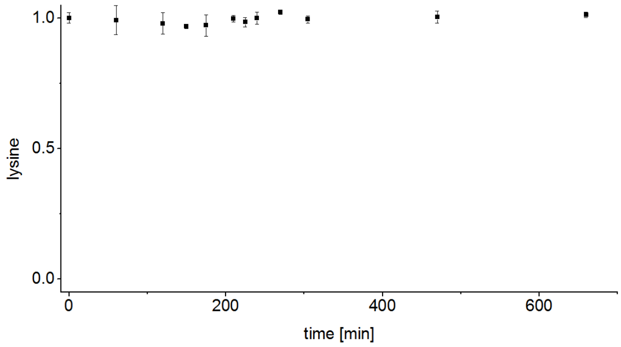
lysine
1.0
0.5
0.0
0
200
400
600
time [min]

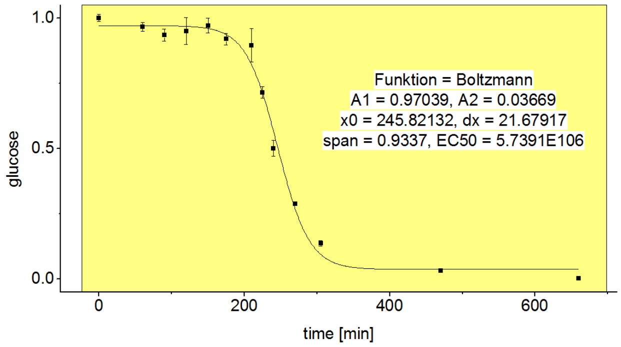
glucose
1.0
0.5
0.0
0
200
400
600
time [min]
Funktion = Boltzmann
A1 = 0.97039, A2 = 0.03669
x0 = 245.82132, dx = 21.67917
span = 0.9337, EC50 = 5.7391E106

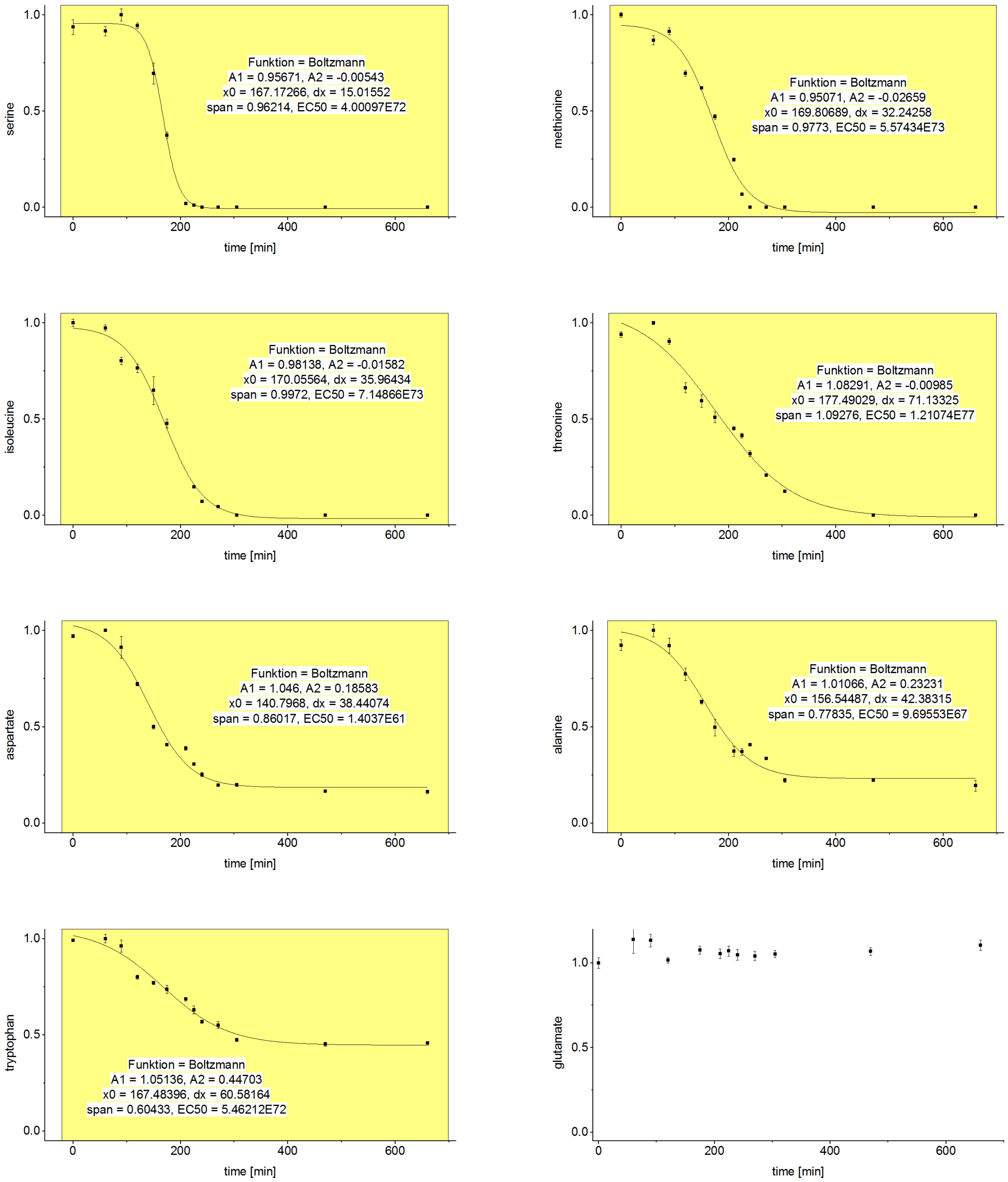

Additional file 1: Substrate consumption in CDMM.
Plotted were relative abundances based on peak areas (except glucose) were obtained by GC-MS or concentrations obtained by enzymatic assays (glucose) and fitted using OriginPro 9.0G software.
