# Supplementary material for: Time-resolved amino acid uptake of Clostridium difficile 630Δerm and concomitant fermentation product and toxin formation
Source: BMC Microbiol. 2015 Dec 18;15:281. doi: 10.1186/s12866-015-0614-2 (PMC4683695; doi:10.1186/s12866-015-0614-2)
Supplement: Additional file 2: — Substrate consumption and product formation in MDM. Plotted were relative abundances based on peak areas (except glucose, formate and ammonium) were obtained by GC-MS or concentrations obtained by enzymatic assays (glucose, formate and ammonium) and fitted using OriginPro 9.0G software. (PDF 371 kb) [file 12866_2015_614_MOESM2_ESM.pdf]

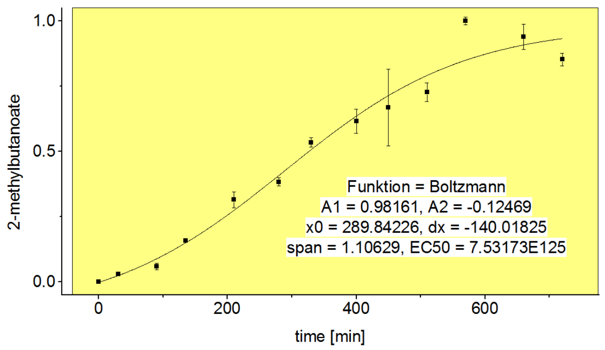
2-methylbutanoate
time [min]
Funktion = Boltzmann
A1 = 0.98161, A2 = -0.12469
x0 = 289.84226, dx = -140.01825
span = 1.10629, EC50 = 7.53173E125

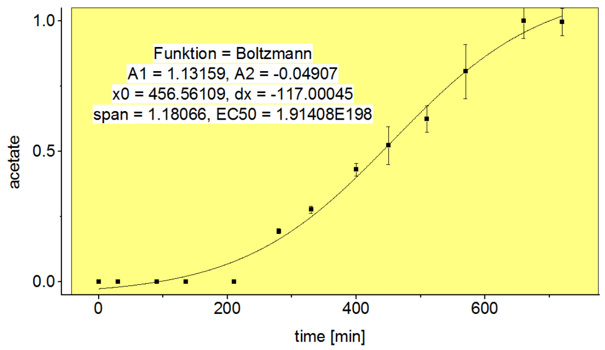
acetate
time [min]
Funktion = Boltzmann
A1 = 1.13159, A2 = -0.04907
x0 = 456.56109, dx = -117.00045
span = 1.18066, EC50 = 1.91408E198

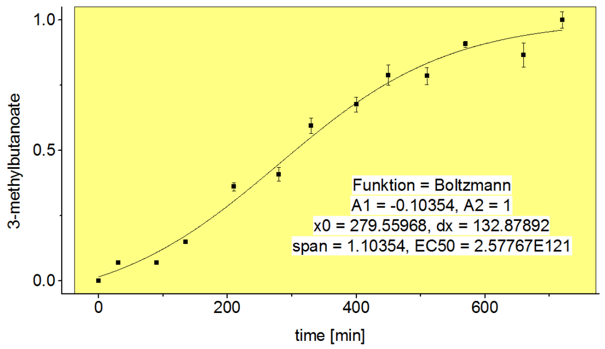
3-methylbutanoate
time [min]
Funktion = Boltzmann
A1 = -0.10354, A2 = 1
x0 = 279.55968, dx = 132.87892
span = 1.10354, EC50 = 2.57767E121

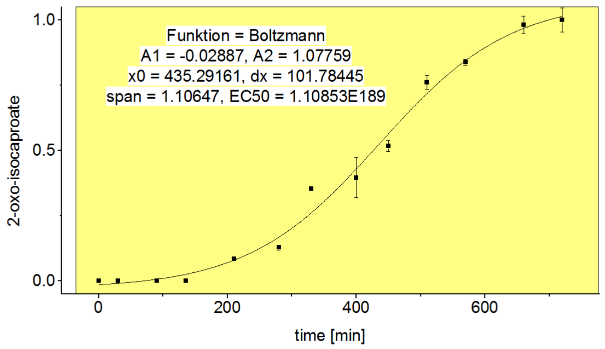
2-oxo-isocaproate
time [min]
Funktion = Boltzmann
A1 = -0.02887, A2 = 1.07759
x0 = 435.29161, dx = 101.78445
span = 1.10647, EC50 = 1.10853E189

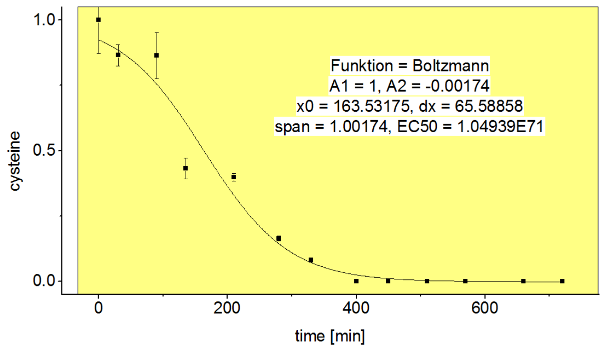
cysteine
time [min]
Funktion = Boltzmann
A1 = 1, A2 = -0.00174
x0 = 163.53175, dx = 65.58858
span = 1.00174, EC50 = 1.04939E71

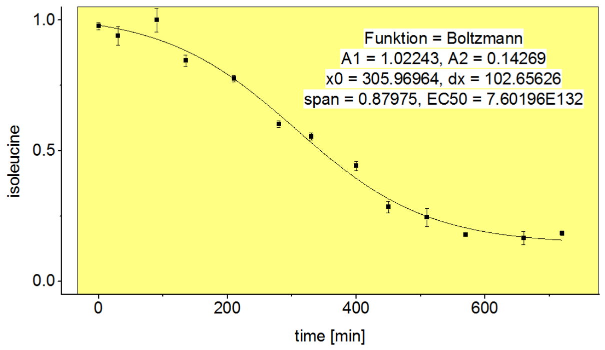
isoleucine
time [min]
Funktion = Boltzmann
A1 = 1.02243, A2 = 0.14269
x0 = 305.96964, dx = 102.65626
span = 0.87975, EC50 = 7.60196E132

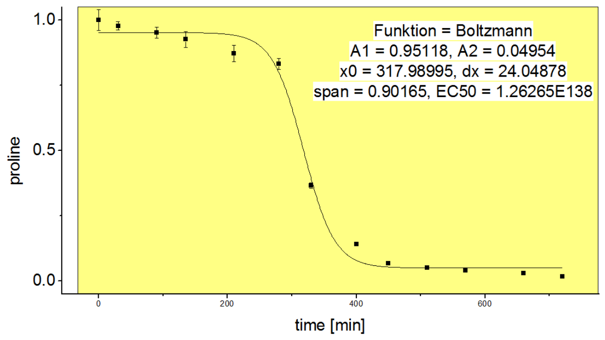
proline
time [min]
Funktion = Boltzmann
A1 = 0.95118, A2 = 0.04954
x0 = 317.98995, dx = 24.04878
span = 0.90165, EC50 = 1.26265E138

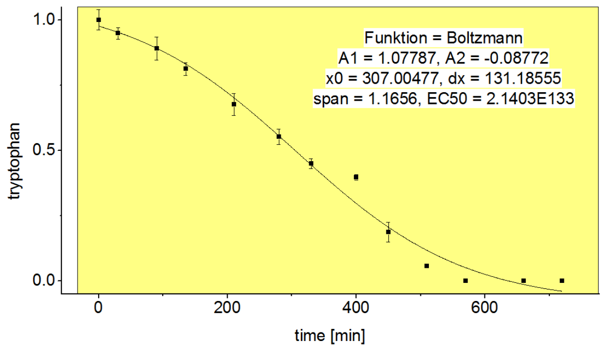
tryptophan
time [min]
Funktion = Boltzmann
A1 = 1.07787, A2 = -0.08772
x0 = 307.00477, dx = 131.18555
span = 1.1656, EC50 = 2.1403E133

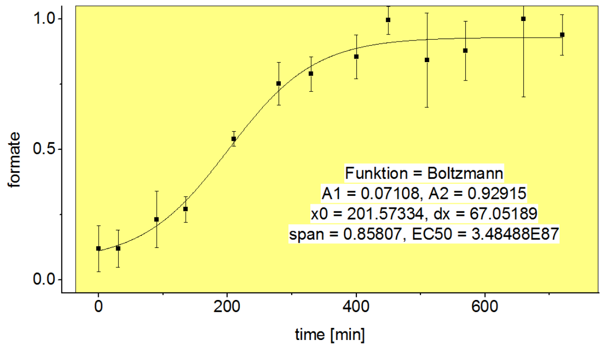
formate
time [min]
Funktion = Boltzmann
A1 = 0.07108, A2 = 0.92915
x0 = 201.57334, dx = 67.05189
span = 0.85807, EC50 = 3.48488E87

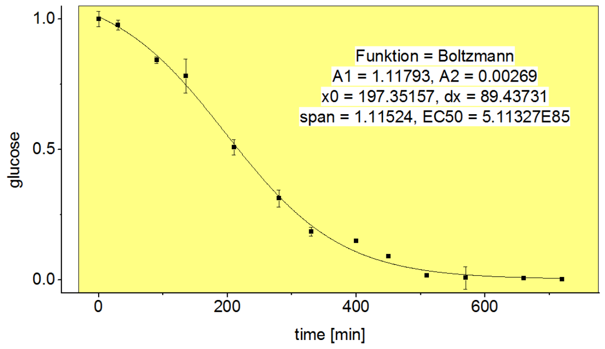
glucose
time [min]
Funktion = Boltzmann
A1 = 1.11793, A2 = 0.00269
x0 = 197.35157, dx = 89.43731
span = 1.11524, EC50 = 5.11327E85

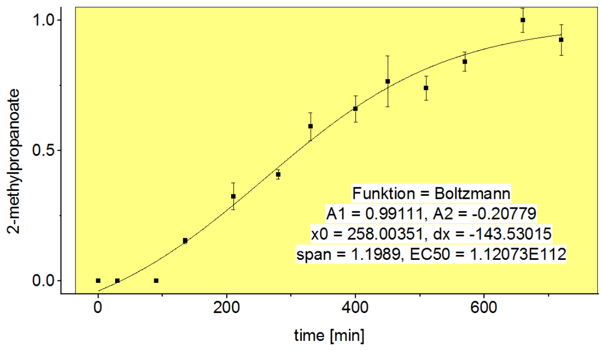

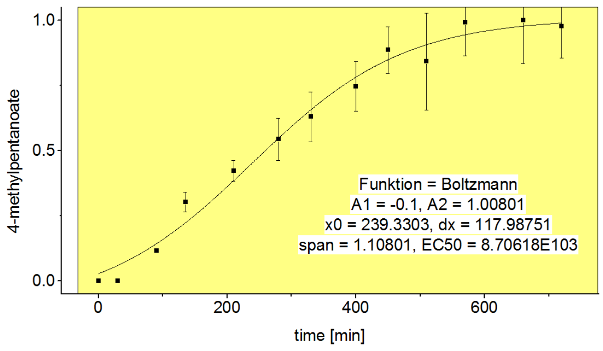

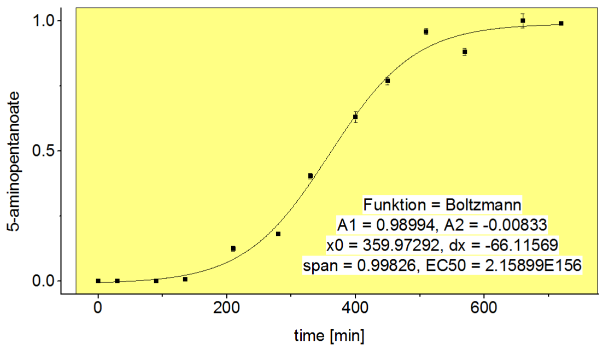

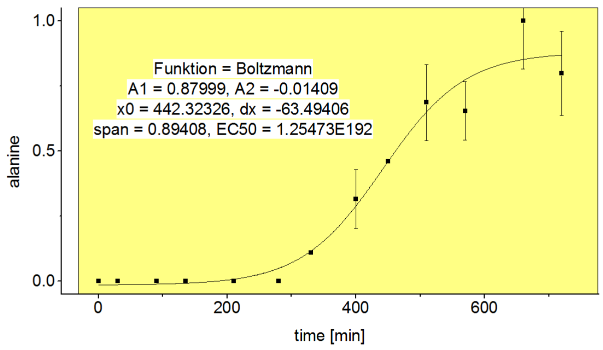

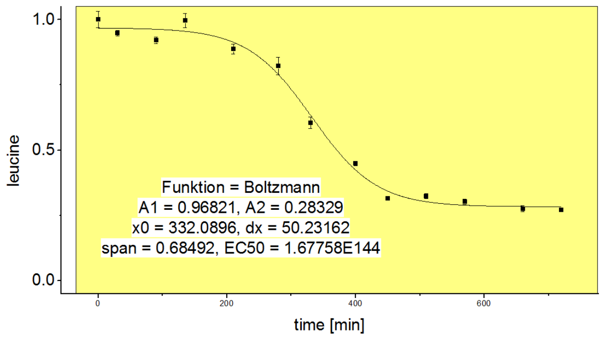

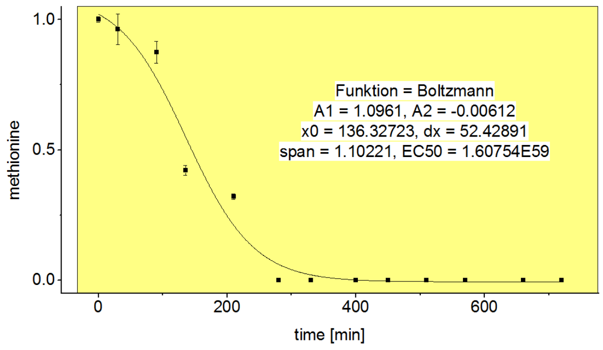

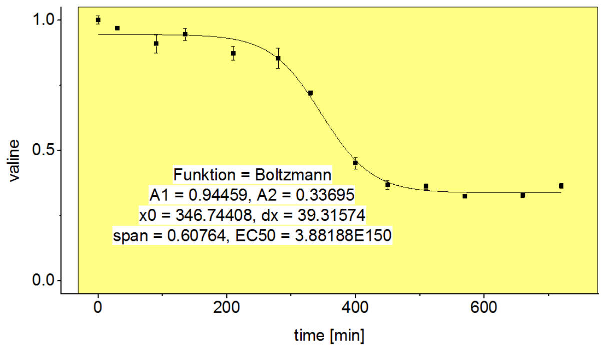

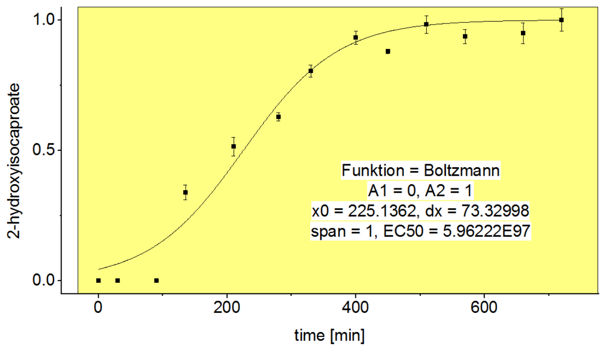

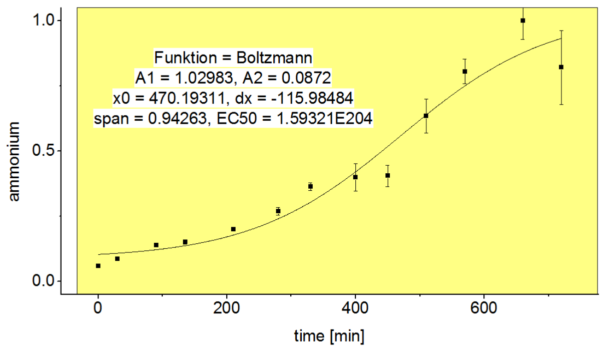

Additional file 2: Substrate consumption and product formation in MDM. Plotted were relative abundances based on peak areas except (glucose, formate and ammonium) were obtained by GC-MS or concentrations obtained by enzymatic assays (glucose, formate and ammonium) and fitted using OriginPro 9.0G software.
