# Supplementary material for: Time-resolved amino acid uptake of Clostridium difficile 630Δerm and concomitant fermentation product and toxin formation
Source: BMC Microbiol. 2015 Dec 18;15:281. doi: 10.1186/s12866-015-0614-2 (PMC4683695; doi:10.1186/s12866-015-0614-2)
Supplement: Additional file 3: — Product formation in CDMM. Plotted were relative abundances based on peak areas (except formate and ammonium) were obtained by GC-MS or concentrations obtained by enzymatic assays (formate and ammonium) and fitted using OriginPro 9.0G software. (PDF 397 kb) [file 12866_2015_614_MOESM3_ESM.pdf]

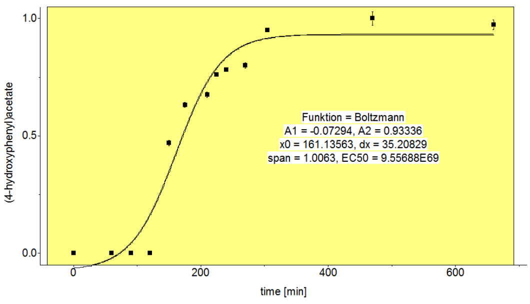
(4-hydroxyphenyl)acetate
Funktion = Boltzmann
A1 = -0.07294, A2 = 0.93336
x0 = 161.13563, dx = 35.20829
span = 1.0063, EC50 = 9.55688E69
time [min]

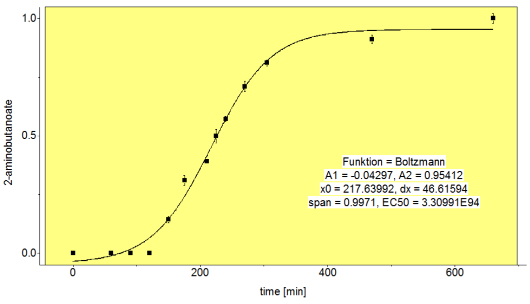
2-aminobutanoate
Funktion = Boltzmann
A1 = -0.04297, A2 = 0.95412
x0 = 217.63992, dx = 46.61594
span = 0.9971, EC50 = 3.30991E94
time [min]

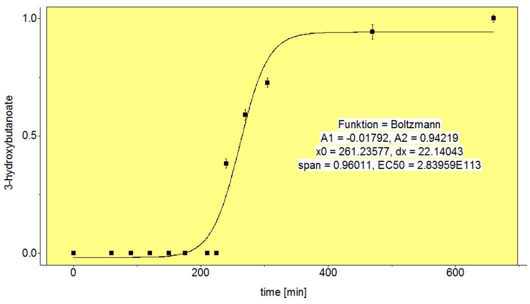
3-hydroxybutanoate
Funktion = Boltzmann
A1 = -0.01792, A2 = 0.94219
x0 = 261.23577, dx = 22.14043
span = 0.96011, EC50 = 2.83959E113
time [min]

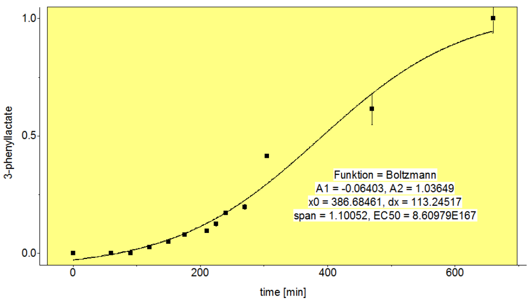
3-phenyllactate
Funktion = Boltzmann
A1 = -0.06403, A2 = 1.03649
x0 = 386.68461, dx = 113.24517
span = 1.10052, EC50 = 8.60979E167
time [min]

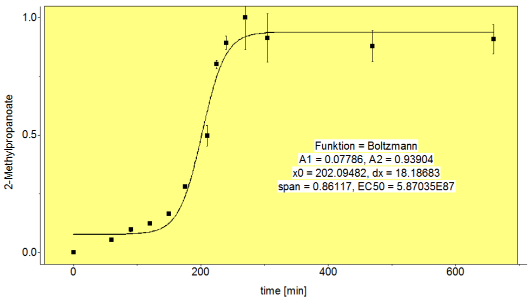
2-Methylpropanoate
Funktion = Boltzmann
A1 = 0.07786, A2 = 0.93904
x0 = 202.09482, dx = 18.18683
span = 0.86117, EC50 = 5.87035E87
time [min]

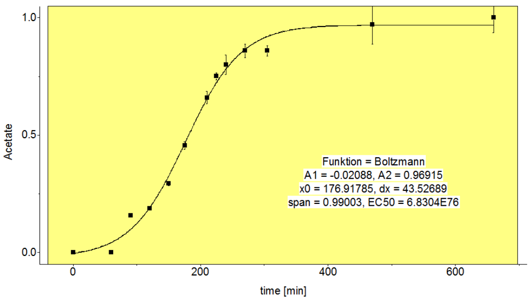
Acetate
Funktion = Boltzmann
A1 = -0.02088, A2 = 0.96915
x0 = 176.91785, dx = 43.52689
span = 0.99003, EC50 = 6.8304E76
time [min]

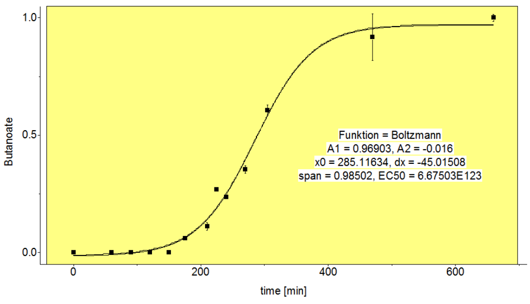
Butanoate
Funktion = Boltzmann
A1 = 0.96903, A2 = -0.016
x0 = 285.11634, dx = -45.01508
span = 0.98502, EC50 = 6.67503E123
time [min]

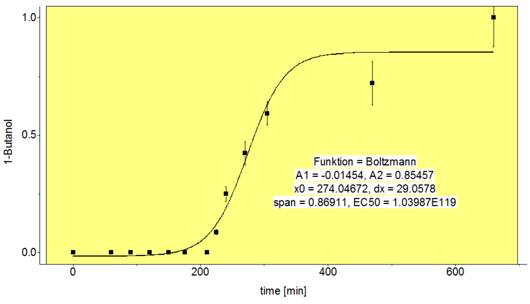
1-Butanol
Funktion = Boltzmann
A1 = -0.01454, A2 = 0.85457
x0 = 274.04672, dx = 29.0578
span = 0.86911, EC50 = 1.03987E119
time [min]

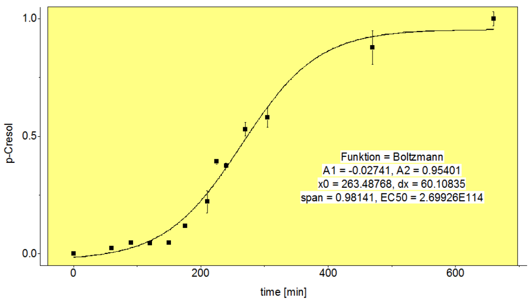
p-Cresol
Funktion = Boltzmann
A1 = -0.02741, A2 = 0.95401
x0 = 263.48768, dx = 60.10835
span = 0.98141, EC50 = 2.69926E114
time [min]

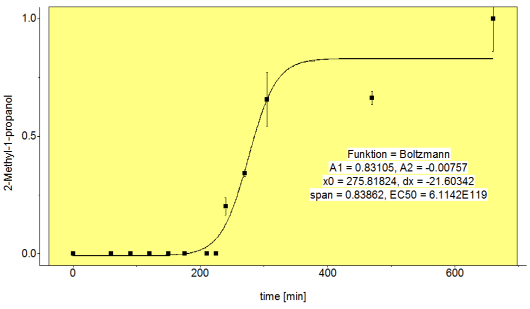
2-Methyl-1-propanol
Funktion = Boltzmann
A1 = 0.83105, A2 = -0.00757
x0 = 275.81824, dx = -21.60342
span = 0.83862, EC50 = 6.1142E119
time [min]

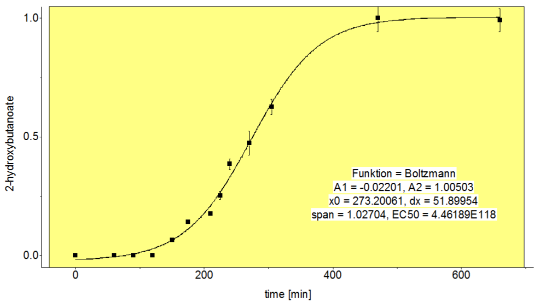
2-hydroxybutanoate
time [min]
Funktion = Boltzmann
A1 = -0.02201, A2 = 1.00503
x0 = 273.20061, dx = 51.89954
span = 1.02704, EC50 = 4.46189E118

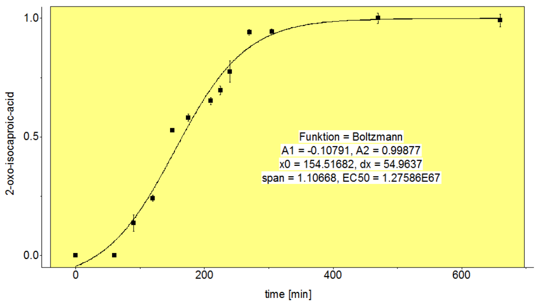
2-oxo-isocaproic-acid
time [min]
Funktion = Boltzmann
A1 = -0.10791, A2 = 0.99877
x0 = 154.51682, dx = 54.9637
span = 1.10668, EC50 = 1.27586E67

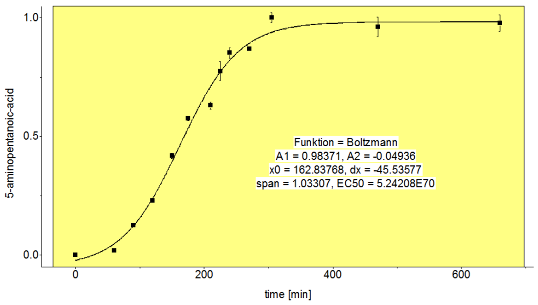
5-aminopentanoic-acid
time [min]
Funktion = Boltzmann
A1 = 0.98371, A2 = -0.04936
x0 = 162.83768, dx = -45.53577
span = 1.03307, EC50 = 5.24208E70

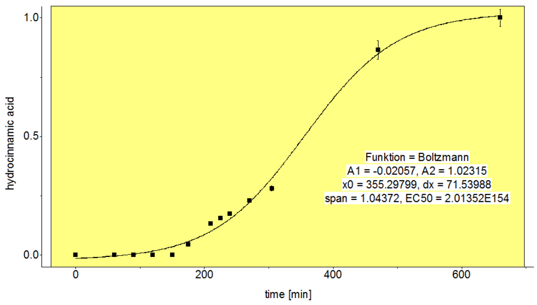
hydrocinnamic acid
time [min]
Funktion = Boltzmann
A1 = -0.02057, A2 = 1.02315
x0 = 355.29799, dx = 71.53988
span = 1.04372, EC50 = 2.01352E154

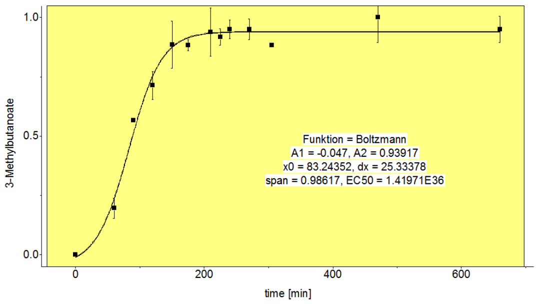
3-Methylbutanoate
time [min]
Funktion = Boltzmann
A1 = -0.047, A2 = 0.93917
x0 = 83.24352, dx = 25.33378
span = 0.98617, EC50 = 1.41971E36

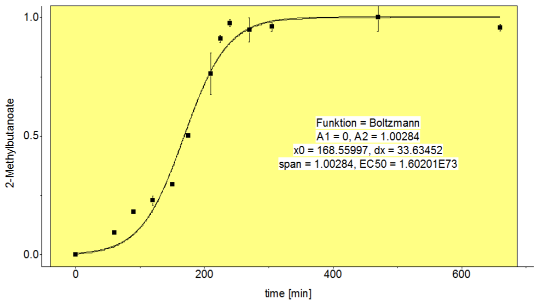
2-Methylbutanoate
time [min]
Funktion = Boltzmann
A1 = 0, A2 = 1.00284
x0 = 168.55997, dx = 33.63452
span = 1.00284, EC50 = 1.60201E73

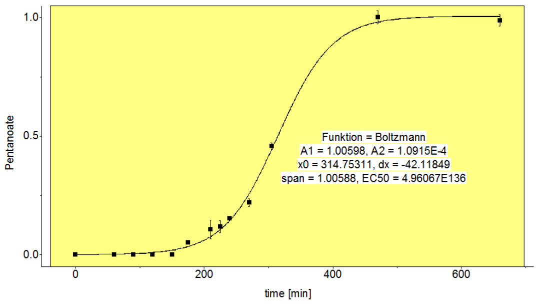
Pentanoate
time [min]
Funktion = Boltzmann
A1 = 1.00598, A2 = 1.0915E-4
x0 = 314.75311, dx = -42.11849
span = 1.00588, EC50 = 4.96067E136

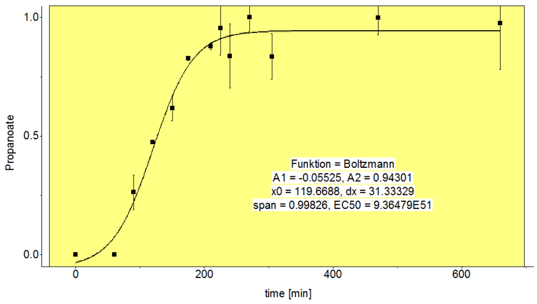
Propanoate
time [min]
Funktion = Boltzmann
A1 = -0.05525, A2 = 0.94301
x0 = 119.6688, dx = 31.33329
span = 0.99826, EC50 = 9.36479E51

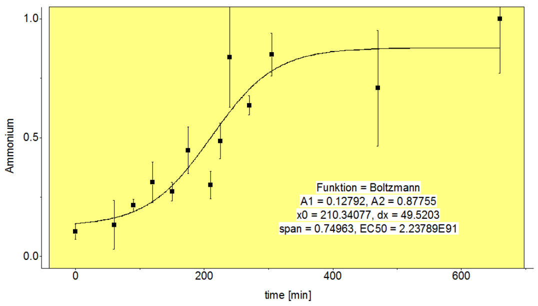
Ammonium
time [min]
Funktion = Boltzmann
A1 = 0.12792, A2 = 0.87755
x0 = 210.34077, dx = 49.5203
span = 0.74963, EC50 = 2.23789E91

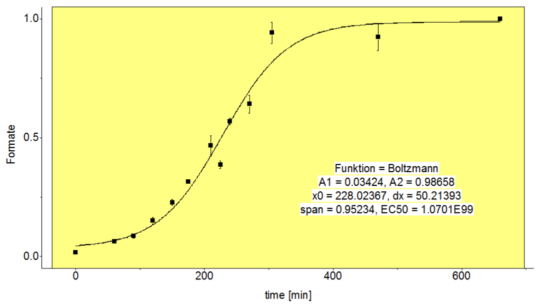
Formate
time [min]
Funktion = Boltzmann
A1 = 0.03424, A2 = 0.98658
x0 = 228.02367, dx = 50.21393
span = 0.95234, EC50 = 1.0701E99

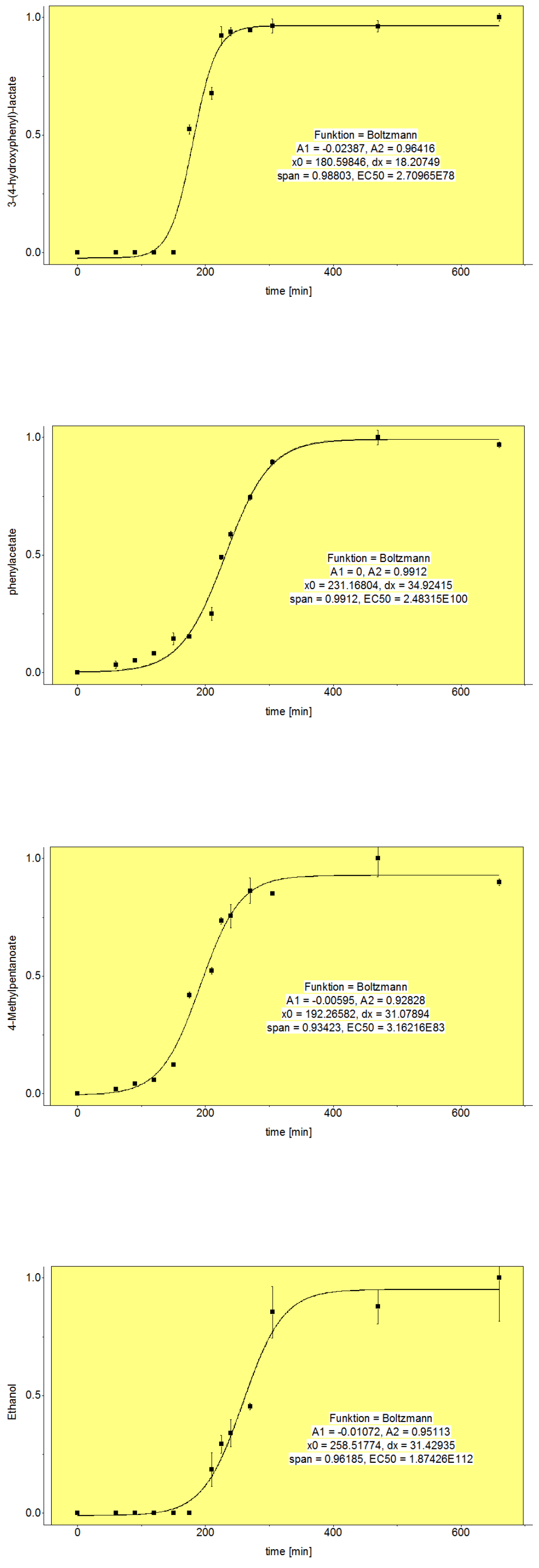

Additional file 3: Product formation in CDMM. Plotted were relative abundances based on peak areas (except formate and ammonium) were obtained by GC-MS or concentrations obtained by enzymatic assays (formate and ammonium) and fitted using OriginPro 9.0G software.
