# Supplementary material for: Time-resolved amino acid uptake of Clostridium difficile 630Δerm and concomitant fermentation product and toxin formation
Source: BMC Microbiol. 2015 Dec 18;15:281. doi: 10.1186/s12866-015-0614-2 (PMC4683695; doi:10.1186/s12866-015-0614-2)
Supplement: Additional file 4: — Casein composition and deduced amino acid composition of casamino acids. Data were confirmed by enzymatic measurements of alanine, glutamate and free amino acid contents. (PDF 34 kb) [file 12866_2015_614_MOESM4_ESM.pdf]

Additional file 4: **Casein composition and deduced amino acid composition of casamino acids.** Data were confirmed by enzymatic measurements of alanine, glutamate and free amino acid contents.

amino acid (aa)	number of amino acids (protein sequence)					aa/aa_{total}			aa/casein	aa/alanine
	casein subunit (bovine)				SUM	casein	hydrolyzed			
	α-S1	α-S2	β	κ		%	%	g/mol	%	%
alanine	12	11	9	16	48	6	6	5.03	4	100
arginine	6	6	4	5	21	2	2	4.30	3	86
asparagine	8	14	5	8	35	4	0	0.00	-	-
aspartate	7	4	4	4	19	2	6	8.46	7	168
cysteine	1	3	1	2	7	1	0	0.00	-	-
glutamate	25	24	19	12	80	9	17	25.10	19	499
glutamine	14	16	20	15	65	8	0	0.00	-	-
glycine	9	2	5	3	19	2	2	1.68	1	33
histidine	5	3	5	3	16	2	2	2.92	2	58
lsoleucine	12	12	11	13	48	6	6	7.41	6	147
leucine	22	16	27	13	78	9	9	12.04	9	239
lysine	15	25	12	10	62	7	7	10.66	8	212
methionine	6	5	7	4	22	3	3	3.86	3	77
phenylalanine	8	9	9	7	33	4	4	6.41	5	127
proline	17	10	35	21	83	10	10	11.24	9	223
serine	16	17	16	14	63	7	7	7.79	6	155
threonine	6	16	9	17	48	6	6	6.73	5	134
tryptophan	2	2	1	1	6	1	0	0.00	-	-
tyrosine	10	12	4	9	35	4	4	7.46	6	148
valine	13	15	21	13	62	7	7	8.55	7	170
SUM	214	222	224	190	850	100	98	129.64	100	2576
